# Bioactive glass granules S53P4 in osteotomy Le Fort I

**DOI:** 10.1038/s41598-020-68932-0

**Published:** 2020-07-29

**Authors:** Eduardo Luis de Souza Cruz, Fernando Jordão de Souza, Lucas Machado de Menezes, Fabrício Mesquita Tuji, José Thiers Carneiro

**Affiliations:** 1 Dentistry Post-Graduation Program of Federal, University of Pará, Augusto Correa Street, 01, Belém, PA 66075-110 Brazil; 2 Ophir Loyola Hospital, Governador Magalhães Barata Avenue, 992, Belém, PA 66063-240 Brazil

**Keywords:** Osteoblasts, Oral anatomy

## Abstract

We evaluated bioactive glass graft (S53P4) in patients undergoing Le Fort I osteotomy, with non-grafted patients as controls. Computed tomography facial scans of the 25 patients submitted for Le Fort I were divided into two groups: Group 1—S53P4 group and Group 2—without grafting. CT scans were analyzed in the immediate postoperative period (T1) and 6 months later (T2), for linear bone gap measurements, tomographic radiodensity and behavior of the maxillary sinus. A Kruskal–Wallis test on bone gap data adopted α significance levels (*p* ≤ 0.05). The Friedman test (*p* ≤ 0.05) was used to evaluate sinus reaction cores. For gap measurements, we observed a decrease in median data between T1 and T2 in both groups, with statistical significances observed between groups in T0; G1 presented statistical difference in its two studied times (*p* ≤ 0.0001). For bone density, the studied data behaved inversely. G1′s bone density decreased from T1 to T2, whereas in G2 there was an increase from T1 to T2. S53P4 did not elicit increased reactions and/or sinus infections in the G1 group (*p* ≥ 1.00). S53P4 did not impact on Le Fort I osteotomies as a coadjuvant and a favorable factor in bone formation, and appeared innocuous in the maxillary sinus.

## Introduction

Orthognathic surgery mobilizes and repositions the jaw bone base via osteotomy, to ensure functional and aesthetic results for dental-skeletal discrepancies^1–3^. Tridimensional surgical movements of the upper jaw are considered unstable with larger bone gaps, thus increasing the need for bone grafts, especially in upper jaw advances of > 4 mm, which normally generate bone discontinuities of > 3 mm^1–7^.

Autogenous grafts minimize large bone discontinuities in the osteotomy line^4,6–8^. Similarly, several bone substitutes have been created to decrease morbidity in patients^9–11^. This follows recent technological innovations that cater for increased tissue regeneration and pathogen inhibition^12-14^.

In 1969, Hench et al. developed materials based on bioactive glass^12,13^. Since then, these materials have been used in modern clinical applications such as dentistry, orthopedic surgeries and otorhinolaryngology^12^. S53P4 produces osteoconductive biological responses at tissue-biomaterial interfaces, by forming silica gel layers that attract and differentiate osteoblasts into bone. The material interacts with growth factors to stimulate angiogenesis and pathogen inhibition via elevated pH and osmotic pressure^12,13,15–17^.

No studies have yet explored S53P4 in linear bone defects of few walls as in Le Fort I osteotomies, considering the anatomical proximity to the maxillary sinus and its behavior in front of bioactive glass. Therefore, we evaluated S53P4 in patients undergoing Le Fort I osteotomies, with non-grafted patients as controls.

## Materials and methods

This retrospective observational case–control study evaluated data from medical records and CT scans of individuals who underwent Le Fort I osteotomy using a bioactive glass graft, when compared with non-grafted individuals.

### Ethical considerations

The ethics committee of Ophir Loyola Hospital, Belém, Pará (PA), Brazil approved this study. The reference umber was 2.462.515/2017. All patients were anonymized and signed the Informed Consent Term (ICT).

### Patient sample

Eleven patients who submitted to orthognathic surgery, and grafted with bioactive glass granules between 2015 and 2018 were included. Fourteen un-grafted patients in the same period acted as a control group. In all, 25 records were analyzed and divided into two groups: G1—grafted group (n = 11) and G2—non grafted group (n = 14).

Dental-skeletal discrepancies requiring maxillary advancement of at least 4 mm, requiring Le Fort I osteotomy was the main inclusion criteria. Patients with incomplete records, cases where other graft biomaterials were used, syndromic patients, patients with palatal cleft lip, and individuals with a history of facial skull trauma, sinusitis and infection were excluded.

Variables such as gap size and tomographic density were used to assess the osteoconductive behavior of the biomaterial, while tomographic aspects of the maxillary sinus were assessed for its reactivity to S53P4.

### Surgical planning

All patients were orthopedically prepared under the “Surgery First Approach” philosophy, following appropriate orthodontic protocols. Multi-slice Face Computed Tomography (CT) was generated with a mean dose of 1834 (CTD/Vol 42.20; DLP 1,039.10), into 0.5 mm sections. Pre-operative DICOM imaging (Digital Imaging and Communications in Medicine) was processed by Dolphin Imaging software version 11.7/1988-2014 (Petterson Dental Supply ©, Canada, CA) for virtual planning and custom guides for maxillary advancement.

### Surgical techniques

All patients underwent orthognathic surgery under general anesthesia. Le Fort I osteotomies were similarly performed in all patients. Firstly, a bilateral vestibular-maxillary access was performed with straight dissecting tips (Traumec Health Technology ©, São Paulo, Brazil). In each case, muco-periosteal detachment continued until the region near the frontal process of the maxilla and zygoma body, just as the nasal mucosa was delicately released and protected. Using micro saws (Microaire ©, Rio de Janeiro, Brazil), an osteotomy was performed and finished with surgical chisels. The upper jaws were mobilized and repositioned with surgical customized guides. Fixation was performed with 4 L (right and left) Arnett type plates (KLS Martin Ltd, São Paulo, Brazil), following the principles of the Rigid Internal Fixation—FIR (AO Foundation). Syringes containing 2.5 cc bioactive glass S53P4 Putty (BonAlive Biomaterials Ltd., Turku, Finland) were applied to inter-segmental bone gaps, and adaptations to surfaces were digitally performed. Surgical wounds were closed with scalloped continuous sutures, performed with simple catgut surgical thread.

### Bioactive glass Putty S53P4

S53P4 is a fully synthetic bone substitute, classified as bioactive glass. It acts as an osteo-conductor and osteo-stimulatory agent to promote bone formation through osteoblast recruitment and activation. The material also inhibits bacterial growth by increasing pH and osmotic pressure. BonAlive putty consists of 53% SiO_2_, 23% Na_2_O, 20% CaO and 4% P_2_O_5_ and a synthetic binder—a mixture of polyethylene glycol (PEG) and glycerol. Commercially available 2.5 cc syringe putty (sterile and ready-to-use paste) was used in this study.

### Data collection

Patient charts were reviewed for age, sex, etiology and surgical planning.

### Analysis of bone gap size

**Figure 1:**
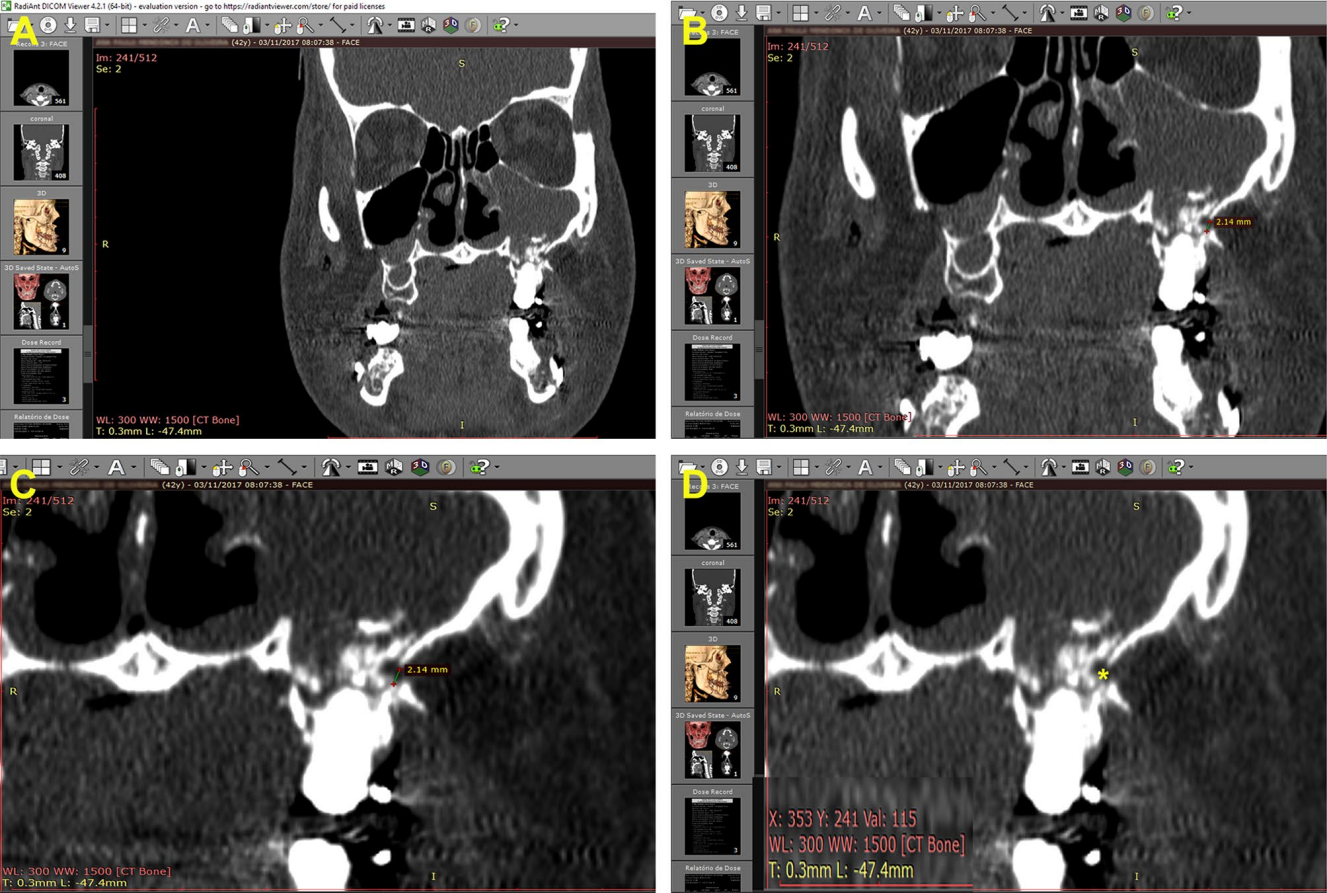
Digital evaluation of bone gaps. (**A**) Visualization of DICOM images in radiant software; (**B**) and (**C**) Linear measurements of bone gaps at lower and higher magnification, respectively; (**D**) X/Y coordinates in the linear center of gaps to measure attenuation coefficients: HU (asterisk)—Software Radiant DICOM Viewer version 4.2.1.17555.

Immediate (T1) and late (T2; 6 months) post-operative face CT scans were analyzed in the Radiant DICOM Viewer software, version 4.2.1.17555 (Medixant, Poznan, Poland) for bone gap measurements in the lateral wall region of the maxillary sinus, between the canine and zygomatic pillars of a randomly chosen side. Measurements were made in triplicate using a “measurements” tool. Mean values were then obtained (Fig. 1).

### Analysis of bone regeneration

For bone regeneration analyses, T1 and T2 tomograms were compared. Parameters included bone attenuation density/coefficients, and followed the Hounsfield Scale^19,20^. To this end, coordinates X/Y were located in the linear half of bone gaps, using the cursor in the 2D function of the Radiant DICOM viewer software (Medixant). This provided integer values compatible with tissue attenuation. The X/Y coordinates were individualized and used as references for the both times of mensuration (Fig. 1).

### Behavior analysis of the maxillar sinus

T1 and T2 CT scans were analyzed using sinusal reaction scores for maxillary sinus behaviors, such as sinus veining and thickening of the lining mucosa: 0—preoperative aspect, normal pneumatization, sinus coating unchanged; 1—immediate postoperative appearance: hemosinus, osteotomies, graft extravasation; 2—presence of sinus reaction: mucous thickening, and 3—presence of sinus reaction and infection.

### Statistical analysis

Data were tabulated and processed using Bioestat 5.0 Software (Mamirauá Institute, Amazon, Brazil) to calculate differences between medians, interquartile deviations and sinus reaction scores, considering the abnormality data of gaps measurements and tomographic radiodensity. For this, the Kruskal–Wallis test was used for independent samples, adopting an α level of significance (*p* ≤ 0.05). Intra-class correlations were used to measure the replicability of triplicate measurements (*p* ≤ 0.01) adopting a 95% confidence interval. For statistical analysis of sinusal behaviors, the Friedman test was used, adopting an α level of significance (*p* ≤ 0.05).

### Ethical approval

All procedures involving human participants were performed in accordance with the ethical standards of the institutional research committee, and with the 1964 Helsinki declaration and its later amendments.

## Results

Our data demonstrated larger gaps in grafted patients (G1), which justified the use of the biomaterial demonstrated by high tomographic density. However, we did not observe more bone formation during the study in grafted patients group. The maxillary sinus aspect remained the same in both groups (Tables 1, 2, 3).

**Table 1:** G1 data.

Patient	Bone gap immediate^A^ (mm)	Immediate radiodensity^C^	Late bone gap^B^ (mm)	Late radiodensity^D^
B	7.33	679	0	979
C	4.24	135	3.16	76*
D	3.74	690	0	990
E	4.36	850	4.27	123
F	5.12	603	3.74	152
G	3.46	498	0	952
K	2.28	153	1.83	43
O	3.4	612	0	850
P	2.64	626	2.31	182
N	2	573	1	283

*Patient who were reoperated during the study.

*Validation of the method in triplicate* Bone gap: Excellent replicability (*p* < 0.0001; Intraclass correlation: 0.9938; 95% CI: 0.9731–0.9986)^A^; (*p* < 0.0001; Intraclass correlation: 0.9844; 95% CI: 0.9329- 0.9964)^B^. Radiodensity: Excellent replicability (*p* < 0.0001; intraclass correlation: 0.9998; 95% CI: 0.9993–10,000)^C^; (*p* < 0.0001; intraclass correlation: 1.0000; 95% CI: 0.9999–10,000)^D^.

**Table 2:** G2 data.

Patient	Bone gap immediate^A^ (mm)	Immediate radiodensity^C^	Late bone gap^B^ (mm)	Late radiodensity^D^
b	4.22	56	1.3	296
c	3.79	25	2.27	72
e	1.5	16	0	876
f	1.6	68	0	931
j	2.2	82	0	994
o	2	76	0	1,262
r	2.45	38	1	516
s	2.22	92	0	971
v	2.8	102	1.44	283
u	2.53	61	0	989
x	2.44	23	0	678
q	1.88	59	0	740
k	2.64	114	1.32	251
l	2	68	0.5	348

*Patient who were reoperated during the study.

*Validation of the method in triplicate* Bone gap: Excellent replicability (*p* < 0.0001; Intraclass correlation: 0.9992; 95% CI: 0.9958–0.9998)^a^; (*p* < 0.0001; Intraclass correlation: 0.9996; 95% CI: 0.9981–0.9999)^b^. Radiodensity: Excellent replicability (*p* < 0.0001; Intraclass correlation: 0.9960; 95% CI: 0.9801–0.9992)^c^; (*p* < 0.0001; Intraclass correlation: 0.9999; 95% CI: 0.9997–1.0000)^d^.

**Table 3:** Behavior of the maxillar sinus in T1 and T2.

Patient (G1)	Immediate scorelate score		Patient (G2)	Immediate scorelate score	
A	1	2	b	1	0
B	1	0	c	1	2
C	1	2	e	1	0
D	1	2	f	1	0
E	1	2	j	1	2
F	1	0	o	1	2
G	1	0	r	1	2
K	1	2	s	1	2
O	1	2	v	1	2
P	1	2	u	1	0
N	1	0	x	1	0
			q	1	2
			k	1	0
			l	1	0

Score: 0—preoperative aspect: breasts with normal pneumatization; 1—immediate postoperative appearance: hemosinus, osteotomies, graft extravasation; 2—presence of sinus reaction: mucous thickening; 3—presence of sinus reaction and infection.

### Sample profile

**Figure 2:**
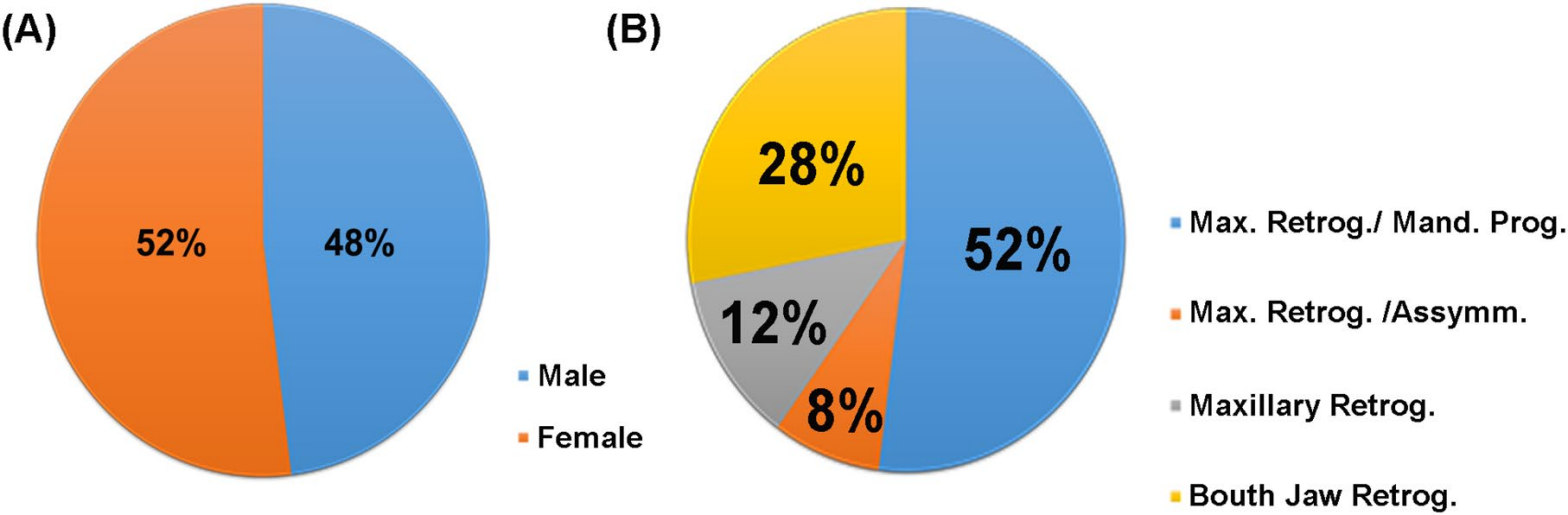
Sample profiles. Descriptive analysis of participant gender (**A**) and etiology (**B**).

The sample consisted of 25 individuals (Table 4), of which 48% (12/25) were male and 52% (13/25) female, with an average age of 29.3 years. The main etiological factors were maxillary retrognatism associated with mandibular prognathism (52%, 13/25), bimaxillary retrognatism (28%, 7/25), maxillary retrognatism (12%, 3/25) and maxillary retrognatism associated with asymmetry (8% , 2/25) (Fig. 2).

**Table 4:** Sample profile.

G1					G2				
Patient	Sex	Age (years)	Etiology	Mean maxillary advanc. (mm)	Patient	Sex	Age (years)	Etiology	Mean maxillary advanc. (mm)
A	Male	31	Max. Retrog./Mand.Prog	5	a	Female	35	Max. Retrog./Mand.Prog	4
A	Male	31	Max. Retrog./Mand.Prog	5	a	Female	35	Max. Retrog./Mand.Prog	4
B	Male	25	Max. Retrog./Mand.Prog	8	b	Male	34	Max. Retrog./Mand.Prog	8
C*	Female	42	Max. Retrog./Mand.Prog	4	c	Male	18	Maxillary Retrog	4
D	Female	31	Max. Retrog./Assymm	4	d	Female	32	Max. Retrog./Assymm	4
E	Female	37	Max. Retrog./Mand.Prog	5	e	Male	23	Bouth Jaw Retrog	4
F	Male	34	Max. Retrog./Mand.Prog	7	f	Female	39	Bouth Jaw Retrog	6
G	Male	20	Max. Retrog./Mand.Prog	7	g	Female	24	Bouth Jaw Retrog	4
H	Female	28	Max. Retrog./Mand.Prog	4	h	Male	23	Max. Retrog./Mand.Prog	4
I	Female	37	Max. Retrog./Assymm	6	i	Male	30	Bouth Jaw Retrog	5
J	Female	48	Bouth Jaw Retrog	4	j	Female	29	Max. Retrog./Mand.Prog	5
L	Male	20	Bouth Jaw Retrog	4	l	Female	18	Maxillary Retrog	4
					l	Female	18	Maxillary Retrog	4
					m	Female	30	Max. Retrog./Mand.Prog	4
					n	Male	46	Bouth Jaw Retrog	4
					n	Male	30	Max. Retrog./Mand.Prog	4
Mean age (years): 29, 3			Mean maxillary advancement (mm): 4, 88		Male/female: 12/13			Total: 25	

*Patients who were reoperated during the study.

### Gaps bones in post-operative T1/T2

In terms of linear bone gap measurements, G1 had the highest gaps in T1, when compared to the control group. This difference was statistically significant (*p* ≤ 0.0001).

There was a decrease in median gap sizes between T1 and T2 in both G1 and G2 groups, with a statistically significant difference only in G1, with a higher interquartile variance in T2 (*p* ≤ 0.0001).

G2 presented minor interquartile deviations. Measurements showed a tendency to zero in T2, with no statistical differences (Table5).

**Table 5:** Difference between median and interquartile deviation of the bone regeneration analysis through the linear measurement of the bone gaps.

	G1		G2	
	T0	T6	T0	T6
Median	3.740^A,a^	1.830^b^	2.330^B^	0.000
Interquartile deviation	± 1.28	± 3.18	± 0.61	± 1.22
(*p*) ≤ 0.0001				

Kruskal–Wallis for independent samples, adopting α level of significance (*p* ≤ 0.05).

*Upper case letters indicate 5% statistical difference between the different times of analysis of bone gaps intergroups. Lowercase distinct letters indicate statistical difference 5% between the different times of analysis of bone gaps intragroups.

### Bone density in post-operative T1/T2

The groups behaved in reverse about tomographic density in two times studied. In G2, there was a marked increase in tomography density between T1 and T2, while in G1 this magnitude decreased. In both groups, there were significant interquartile deviations in T2.

There was a statistical difference between medians bone density of T1 G1; T1 G2 and T2 G1; T2 G2 (*p* ≤ 0.0002), with G1 approaching zero due to variations in T2. There were no statistical differences in intragroup analyses (Table 6).

**Table 6:** Difference between median and interquartile deviation of the bone regeneration analysis through the tomographic coefficient of tissue attenuation (HU).

	G1		G2	
	T0	T0	T6	T6
Median	612.00^A^	264.00^B^	64.500^C^	709.000^D^
Interquartile deviation	± 117.0	± 763.5	± 38.0	± 652.00
(*p*) ≤ 0.0002				

Kruskal–Wallis for independent samples, adopting α level of significance (*p* ≤ 0.05).

*Upper case letters indicate 5% statistical difference between the different times of analysis of bone gaps intergroups. Lowercase distinct letters indicate statistical difference 5% between the different times of analysis of bone gaps intragroups.

### Sinusal reactions

Verified variables—sinusal reaction aspects—showed no statistical differences (*p* ≥ 1.00) in T1 and T2, in intergroup and intragroup analyses. S53P4 did not generate increased reactions and/or infections in patients, and therefore the null hypothesis was accepted: there is not damage when S53P4 is used (Table 7).

**Table 7:** Reaction analysis of maxillary sinus after inclusion of bone graft. Friedman test, adopting α level of significance (*p* ≤ 0.05).

	G1		G2	
	T0	T1	T0	T1
Median	1	2	1	2
Standard deviation	± 0.00	± 01.50	± 0.00	± 2.00
Total of ranks	31.0	42.0	31.0	36.0
	(*p*) = 0.3189			

*Upper case letters indicate 5% statistical difference between the different times of analysis of bone gaps intergroups. Lowercase distinct letters indicate statistical difference 5% between the different times of analysis of bone gaps intragroups.

## Discussion

Le Fort I osteotomies are indicated for maxillary mobility and repositioning, for the treatment of dento-skeletal discrepancies that affect the middle third of the face. Maxillary advancement and extrusion movements generate inter-segmental bone spaces, with a requirement for bone grafting, proportional to the size of the resulting gaps^4,6–8^. Our study investigated 25 cases of bone gaps requiring bone grafting, in view of the significant surgical maxillary advancement that resulted in the incidence of considerable gaps, especially in G1 (Table 1, 2, 3). We wanted to verify S53P4 applicability to linear defects resulting from Le Fort I.

Factors such as age and sex interfere with tissue repair processes^4,6,7^, however, both groups presented a good gender ratio (male: female 12/13) and were young (mean age 39 years), thus these factors did not appear to exert a strong influence on our data.

Classically, autogenous grafts are indicated for bone regeneration, but with the advent of new biomaterials, other alternatives are viable^4,6–8^. Grafts based on bioactive glass granules are haloplasts materials capable of stimulating osteoblast differentiation and activation via the formation of silica phosphate layers^12–18^. Our study is the first to report S53P4 in Le Fort I osteotomies, for advances > 4 mm. In addition, S53P4 does not require new surgical sites, guaranteeing less surgical time and postoperative morbidity.

Our grafted group showed a gradual decrease in bone gaps at 6 months, indicating bone repair; in non-grafted group, the gaps were lower in T1 and showed a tendency to healing in 6 months. This suggests ideal conditions for new bone formation. In G1, linear measurements were larger, the bone repair did not keep up the G2 data trend, even with the addition of S53P4 that should have minimized differences in bone gaps sizes. In this regard, S53P4 was not ideal for bone repair in our postoperative period.

The Hounsfield Scale (HU) was used to understand bone regeneration. Bone density measurements from X-ray absorption, generate attenuation coefficients used to measure degenerative bone changes, and provide parameters for planning oral rehabilitation with osseointegrated implants^19,20^. For our study, the HU Scale was a measure of bone healing, and demonstrated that S53P4 maintained high tissue attenuation coefficients, without necessarily indicating bone consolidation.

Our grafted group (G1) exhibited expected behaviors: i.e. greater tomographic density in T1 consistent with grafting, and decreased indices reflecting the gradual loss of glass beads and/or replacement by bone matrix in the initial T2 mineralization process. In G2, the low density at T1 increased relative to bone deposition. This was compatible with bone corticalization, which was evident in T2. In our view, the bioactive glass based graft is probably effective for long-term bone stimulation, but up to 6 months, it appeared not to act as an accelerator in bone healing processes.

In Le Fort I osteotomies, bone healing and sinus complication incidences are considerable challenges, especially for large maxillary advancements in regions of canine and zygomatic pillars, as well as lateral borders of the maxillary sinus^21,22^. Haloplastic grafts have demonstrated high success rates in determining the potential risk of infectious complications in the maxillary sinus^12,13,21–24^. S53P4 did not increase the prevalence of sinus reactions and/or infection in our study sample. This was probably due to material adaptability to bone surfaces, gap obliteration, and a lack of toxicity with the sinus mucosa, even at 6 months. Under these conditions, the biomaterial behaved similarly to graft biomaterials in the literature.

In general, bone grafts fill spaces and provide accelerated bone regeneration, ensuring segment stability. On the other hand, filling linear gaps with grafts can be obliterated, limiting the interposition of soft tissue between segments. Our results demonstrated that S53P4 may have prevented tissue infiltration into large bony gaps, due to the biomaterial's obliterative potential in maintaining sites for long periods, guaranteeing conditions that are more favorable^24^. In smaller gaps, obliterating agents do not appear to be necessary.

However, fixation success may directly interfere with the surgical prognosis, given that failures provide mobility that impair correct bone repair^25–27^. Our study presented a case of failure of the fixation system, which led to the need for reoperation. S53P4 was subject to the success of rigid fixation.

Other studies, with longer postoperative periods have proven biomaterial efficacy as accelerators of bone formation and biocompatibility^6–10,12–18,21–29^. S53P4 demonstrated potential as a non-determinant stimulator of bone repair at 6 months, and was biological compatible with maxillary sinus membrane. It requires later histological and tomographic analyses to justify its use in Le Fort I osteotomy.

## Conclusions

S53P4 did not optimally behave as a coadjuvant and favorable factor in bone formation for Le Fort I osteotomy in our study. Therefore, we cannot confirm the biomaterial played any fundamental roles in bone healing; bioactive glass putty is subject to the success of rigid internal fixation systems. In our hands, S53P4 was innocuous to the maxillary sinus.

## References

[1] Khechoyan DY (2013). Orthognathic surgery: general considerations. Semin. Plast. Surg..

[2] Charrier JB (2014). Orthognathic surgery in adults: state of the art. Orthod. Fr..

[3] Friscia M (2017). Complications after orthognathic surgery: our experience on 423 cases. Oral. Maxillofac. Surg..

[4] Ueki K (2011). Assessment of bone healing after Le Fort I osteotomy with 3-dimensional computed tomography. J. Craniomaxillofac. Surg..

[5] Soehard IA (2015). Stability, complications, implant survival and patient satisfaction after Le Fort I osteotomy and interposed bone grafts: follow-up of 5–18 years. Int. J. Oral Maxillofac. Surg..

[6] Guiol J, Schendel SAL (2015). Fort I osteotomies combined with post-operative bone grafts. Rev. Stomatol. Chir. Maxillofac. Chir. Orale.

[7] Zanettini LMS (2017). Bone substitutes in Le Fort I osteotomy to promote bone union and skeletal stability. J. Craniofac. Surg..

[8] Cengiz E (2015). Comparison of autologous and heterologous bone graft stability effects for filling maxillary bone gap after Le Fort I osteotomy. Adv. Clin. Exp. Med..

[9] Rueger JM, Linhart W, Sommerfeldt D (1998). Biologic reactions to calcium phosphate ceramic implantations. results of animal experiments. Orthopade.

[10] Lye KW, Deatherage JR, Waite PD (2008). The use of demineralized bone matrix for grafting during Le Fort I and chin osteotomies: techniques and complications. J. Oral Maxillofac. Surg..

[11] Ngo LT, Bruhn R, Custer B (2013). Risk perception and its role in attitudes toward blood transfusion: a qualitative systematic review. Transfus. Med..

[12] Pérez-Tanoira R (2015). Effects of S53P4 bioactive glass on osteoblastic cell and biomaterial surface interaction. J. Mater. Sci. Mater. Med..

[13] Fernandes JS (2017). Multifunctional bioactive glass and glass-ceramic biomaterials with antibacterial properties for repair and regeneration of bone tissue. Acta Biomater..

[14] Kramer FJ, Baethge C, Swennen G (2004). Intra- and perioperative complications of the Le Fort I osteotomy: a prospective evaluation of 1000 patients. J. Craniofac. Surg..

[15] Välimäki VV, Aro HT (2006). Molecular basis for action of bioactive glasses as bone graft substitute. Scand. J. Surg..

[16] Waselau M (2012). Effects of bioactive glass S53P4 or beta-tricalcium phosphate and bone morphogenetic protein-2 and bone morphogenetic protein-7 on osteogenic differentiation of human adipose stem cells. J. Tissue Eng..

[17] Van Gestel NA (2015). Clinical applications of S53P4 bioactive glass in bone healing and osteomyelitic treatment: a literature review. Biomed. Res Int..

[18] Profeta AC, Huppa C (2016). Bioactive-glass in oral and maxillofacial surgery. Craniomaxillofac. Trauma Reconstr..

[19] Zou D, Li W, Deng C, Du G, Xu N (2018). The use of CT Hounsfield unit values to identify the undiagnosed spinal osteoporosis in patients with lumbar degenerative diseases. Eur. Spine J..

[20] Genisa M, Shuib S, Rsjion ZA, Arief EM, Hermana M (2018). Density estimation based on the Hounsfield unit value of cone beam computed tomography imaging of the jawbone system. Proc. Inst. Mech. Eng. H.

[21] Arun K, Gosain MD (2004). Bioactive glass for bone replacement in craniomaxillofacial reconstruction. Plast. Reconstr. Surg..

[22] Proffit WR, White RP, Sarver DM (2003). Contemporary treatment of dentofacial deformity.

[23] Cottrell DA, Wolford LM (1998). Long-term evaluation of the use of coralline hydroxyapatite in orthognathic surgery. J. Oral Maxillofac. Surg..

[24] Turunen T, Peltola J, Yli-Urpo A, Happonen RP (2004). Bioactive glass granules as a bone adjunctive material in maxillary sinus floor augmentation. Clin. Oral Implants Res..

[25] Hofbauer LC, Henneicke H (2018). β-blockers and bone health. J. Clin. Investig..

[26] Roden RD (2010). Principles of bone grafting. Oral Maxillofac. Surg. Clin. N. Am..

[27] Waite PD, Tejera TJ, Anucul B (1996). The stability of maxillary advancement using Le Fort I osteotomy with and without genial bone grafting. Int. J. Oral Maxillofac. Surg..

[28] Bhatt RA, Rozental TD (2012). Bone graft substitutes. Hand Clin..

[29] Wolford LM, Freitas RZ (1999). Porous block hydroxyapatite as a bone graft substitute in the correction of jaw and craniofacial deformities. BUMC Proc..

